# Rhizosheath: An adaptive root trait to improve plant tolerance to phosphorus and water deficits?

**DOI:** 10.1111/pce.14395

**Published:** 2022-07-25

**Authors:** Mehtab Muhammad Aslam, Joseph K. Karanja, Ian C. Dodd, Muhammad Waseem, Xu Weifeng

**Affiliations:** ^1^ Center for Plant Water‐Use and Nutrition Regulation, College of Resource and Environment Fujian Agriculture and Forestry University Fuzhou Fujian China; ^2^ College of Agriculture Yangzhou University Yangzhou Jiangsu China; ^3^ State Key Laboratory of Agrobiotechnology, School of Life Sciences The Chinese University of Hong Kong Shatin Hong Kong; ^4^ The Lancaster Environment Centre Lancaster University Lancaster UK; ^5^ Department of Botany University of Narowal Narowal Pakistan

**Keywords:** alternate wetting and drying cycles, drought, QTLs, water uptake

## Abstract

Drought and nutrient limitations adversely affect crop yields, with below‐ground traits enhancing crop production in these resource‐poor environments. This review explores the interacting biological, chemical and physical factors that determine rhizosheath (soil adhering to the root system) development, and its influence on plant water uptake and phosphorus acquisition in dry soils. Identification of quantitative trait loci for rhizosheath development indicate it is genetically determined, but the microbial community also directly (polysaccharide exudation) and indirectly (altered root hair development) affect its extent. Plants with longer and denser root hairs had greater rhizosheath development and increased P uptake efficiency. Moreover, enhanced rhizosheath formation maintains contact at the root‐soil interface thereby assisting water uptake from drying soil, consequently improving plant survival in droughted environments. Nevertheless, it can be difficult to determine if rhizosheath development is a cause or consequence of improved plant adaptation to dry and nutrient‐depleted soils. Does rhizosheath development directly enhance plant water and phosphorus use, or do other tolerance mechanisms allow plants to invest more resources in rhizosheath development? Much more work is required on the interacting genetic, physical, biochemical and microbial mechanisms that determine rhizosheath development, to demonstrate that selection for rhizosheath development is a viable crop improvement strategy.

## 1 INTRODUCTION

Climate change has generally increased the frequency and intensity of drought in the world's arable soils, thereby restricting crop yields (Fahad et al., 2017; Potopová et al., 2016). Crop productivity is also influenced by its nutritional status such as phosphorus (P) deficiency, which alters plant physiological functions and limits yields (Bista et al., 2018; Farooq et al., 2021). Drought stress and P deficiency frequently co‐occur since any restriction in transpiration limits the uptake of water and nutrients, and may cause agronomic effects that cannot be predicted from analysis of individual stress factors (Bista et al., 2018; Suzuki et al., 2014; Xia et al., 2020; Xu et al., 2020). Drought exacerbates P deficiency in plants by reducing its uptake from the soil, transport, and redistribution *in planta* (Rouphael et al., 2012), with plant uptake restricted by diffusional limitations and/or limited root growth when fertilizers are surface‐applied in water‐limited environments with drying topsoils (Q. Ma et al., 2009). Moreover, P deficiency intensifies drought responses by decreasing root hydraulic conductance (Fan et al., 2007; Radin & Eidenbock, 1984; Sardans & Peñuelas, 2012). Exogenous P application enhanced maize drought tolerance by enhancing leaf gas exchange and biomass accumulation (Zahoor et al., 2021). Persistent drought during plant growth, largely driven by low precipitation and/or high evapotranspiration rates, ultimately decreases plant water and nutrient status and hence crop productivity (Gutschick & Bassirirad, 2003; Morison & Morecroft, 2006).

Phosphorus deficiency alters P assimilation at the root surface (Xia et al., 2020), root architecture, biomass allocation and inhibits shoot growth (Cho et al., 2021) and seed development, thereby limiting plant productivity. Plants have adopted sophisticated strategies to scavenge inorganic phosphate (Pi) and enhance its acquisition from P‐limited soils (Ticconi et al., 2004). These include enhanced exudation of organic anions and phosphatases, activation of membrane localized P‐transporters, and establishing symbiotic associations with soil microorganisms. Enhanced organic acid exudation improved P uptake of *Medicago sativa* when grown in acidic soil with high aluminium (Al) concentrations (Tesfaye et al., 2001), while *Glycine max* adapts to P deficiency by increasing root malate exudation (Liang et al., 2013). Furthermore, P solubilizing enzymes (phosphatases) might have an important role in P acquisition. For example, growth of an Arabidopsis mutant (*Atpap10*—for a root‐secreted acid phosphatase) was substantially reduced compared to wild‐type (WT) plants when grown on media containing organophosphates (L. Wang et al., 2014). Since some of these strategies incur a metabolic cost to the plants, selecting P‐efficient crops is important to agriculture (Jin et al., 2006; Tariq et al., 2017; H. Zhang, Shi et al., 2020).

The rhizosheath is a root adaptive trait, literally defined as the weight of soil that remains attached to roots on excavation (George et al., 2014; Mccully, 1999), that has been correlated with variation in plant tolerance to water deficit (Hartnett et al., 2013). A clear distinction exists between rhizosheath (the soil that adheres to the root surface on excavation), and rhizosphere, which is often defined as the volume of soil surrounding plant roots influenced by root exudates that stimulate microbial abundance (Mathesius, 2015). There is considerable genetic diversity in the ability of roots to bind soil: with some angiosperm species forming little rhizosheath (e.g., *Allium* spp; radish, *Raphanus sativus*; soybean, *G. max*), but others showing substantial rhizosheath development (e.g., *Dactylis glomerata*; alfalfa, *M. sativa*). Both monocotyledons and eudicotyledons show a similar range of specific rhizosheath weight (the ratio of bound soil mass to root mass) although in the former the order Poales (the grasses) bound more soil than order Commelinales (Brown et al., 2017). The rhizosheath of grass species exposed to drought stress is thicker and more stable, potentially enhancing water uptake efficiency (Rabbi et al., 2018; Watt et al., 1994), and allowing plants to adapt to drought episodes (Y. Zhang, Du, Gui, et al., 2020). Although the rhizosheath has been much studied in desert grasses (Bergmann et al., 2009; Danin, 1996; Hartnett et al., 2013; Othman et al., 2004), relatively few studies have focused on the role of rhizosheath in drought resistance of crop plants (Liu, Chen et al., 2019). In dry soils, rhizosheaths have higher water content than bulk soil, and may substantially contribute to water uptake (North & Nobel, 1997). P deficiency facilitates rhizosheath formation, with barley (*Hordeum vulgare)* genotypes showing 18% larger rhizosheaths under P‐deficient than P‐sufficient conditions (Brown et al., 2012). Soil drying and P deficiency stimulated rhizosheath development of white lupin (*Lupinus albus)* mature cluster roots, thereby enhancing P uptake (Aslam, Karanja et al., 2021). Moreover, rhizosheath mass was positively correlated with P uptake under dry conditions (Aslam, Karanja et al., 2021; George et al., 2014), suggesting that the rhizosheath is a pivotal root trait to enhance crop nutrient status and improve drought tolerance via various mechanisms.

**Figure 1:**
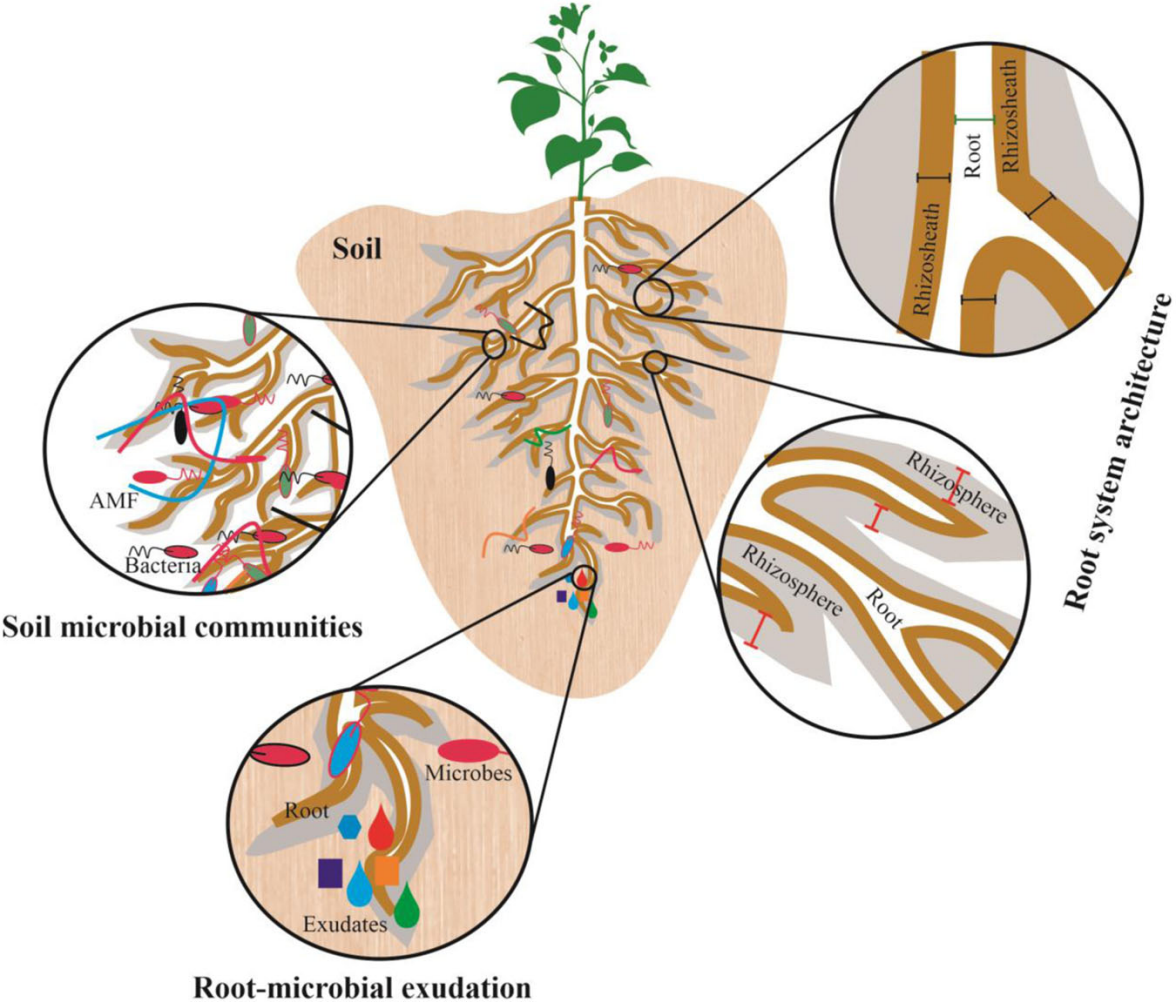
Plant‐soil interactions. The rhizosheath comprises soil particles that adhere to root surface on excavation (shown as brown line). The rhizosphere is the area around plant roots influenced by root exudation and comprising its own microbial community (shown as a grey background) that is distinct from the bulk soil. [Color figure can be viewed at wileyonlinelibrary.com]

Rhizosheath formation involves complex interactions of multiple factors in the rhizosphere (Figure 1), the region surrounded by plant roots and whose microbial activity is highly influenced by root processes (Pang et al., 2017). These factors include root hair traits (Haling et al., 2010), plant root and microbial‐derived mucilage (Carminati et al., 2017; Liu, Ye et al., 2019; Y. Zhang, Du, Xu et al., 2020), microbial activity (Hanna et al., 2013), and soil texture (Haling et al., 2014; Liu, Ye et al., 2019). Physical (root hair length and density) and chemical (root exudates) mechanisms determine rhizosheath development by enmeshing and (dis‐) aggregating soil particles, respectively. Discriminating these mechanisms is difficult since root hairs can be a source of polysaccharides (Galloway et al., 2020) that bind soil particles more effectively than simpler monosaccharide molecules (Morel et al., 1991). While limited rhizosheath development of mutants (lacking root hairs) compared to WT plants demonstrates the importance of root hairs in many crop species (Burak et al., 2021), the importance of root hair traits in modifying rhizosheath development of WT plants varied. In a species (pearl millet—*Pennisetum glaucum*) with relatively short root hairs (<0.7 mm), root hair length was weakly correlated (*r* ^2^ = 0.05) with rhizosheath development (De la fuente Cantó et al., 2022), but in barley with longer root hairs (0.6−2.5 mm), root hair length was slightly better correlated (*r* ^2^ = 0.16) with rhizosheath development(George et al., 2014). Thus species vary in the importance of root hair traits in determining rhizosheath development, probably mediated by the physical dimensions of root hairs and exudate chemistry.

Furthermore, it can be difficult to dissociate plant and microbial mechanisms facilitating rhizosheath development as they can be interdependent. For example, root hairs increased axial and radial rhizosphere extension (Holz et al., 2017), which probably fosters interactions with rhizosheath‐associated microbes. Root traits, most likely rhizodeposition and root hairs, are important to trap more carbon and nutrient resources in the rhizosheath, and attract a healthy microbiome (Hallett et al., 2022). Importantly, root hair growth is finely controlled by multiple plant hormones that are modulated by drought, including abscisic acid (ABA), ethylene, auxins, and cytokinins (Gruber et al., 2013; Vissenberg et al., 2020), which thereby indirectly regulate rhizosheath formation. Moreover, microbial production and degradation of these plant hormones (Dodd et al., 2010) alters root hair development (Contesto et al., 2008), potentially affecting rhizosheath formation.

This review considers key factors that contribute to rhizosheath formation including plant genetics, the microbial community, and physiological mechanisms; then describes how they influence P status in drought‐stressed environments. We also highlight specific mechanisms responsible for improving water and P availability in various crop species and finally suggest areas for further research.

## 2 MECHANISMS OF RHIZOSHEATH FORMATION

### 2.1 Genetics

Plants employ many molecular mechanisms to confer resistance against multiple environmental stresses of both abiotic and/or biotic origin that influence nutrient availability. Many adaptive strategies are attributed to root traits and plasticity since roots sense below‐ground stresses before other plant parts (Passioura, 1988; Zheng et al., 2016), and alter their gene expression for appropriate adaptive responses (Pan et al., 2012). For instance, soil drying regulates expression of various water deprivation‐responsive genes in the roots including pathogenesis‐related protein 4 (PR 4), protein phosphatase 2C (PP 2C), ABA 8′‐hydroxylase, and AP2‐like ER TF (Karanja et al., 2021). Nonetheless, the genetics of crucial traits affecting the rhizosheath remain elusive. Quantitative trait loci (QTLs) analysis in wheat (*Triticum aestivum*) revealed multiple loci (2B, 4D, 5A, 5B, 6A and 7A), which harboured potential basic helix‐loop‐helix (bHLHs) transcription factor genes associated with a robust rhizosheath. For instance, rhizosheath weight of wheat was strongly positively correlated with root hair development (Delhaize et al., 2015). bHLHs also regulate root hair length in *Oryza sativa* and *Arabidopsis thaliana* (Ding et al., 2009; Yi et al., 2010), suggesting a candidate gene underlying a rhizosheath QTL in these species. Thus, rhizosheath formation can be modulated by root hair elongation signalling via these transcription factors.

This model was reinforced by similar studies on foxtail millet (*Setaria italica*) (Liu , Ye et al., 2019), which identified QTLs associated with root hair extension, elongation, and lateral root branching located at the locus that best associated with larger rhizosheath size. In this case, five loci in *S. italica* (*Seita.3G196500, Seita.2G057800, Seita.9G333500, Seita.8G104600* and *Seita.7G190800*) were highly upregulated under drought stress thus hinting at their involvement in rhizosheath formation (Liu, Ye et al., 2019). Collectively, the identification of several loci in the genomes of crop species that affect rhizosheath weight suggests that multiple genes are responsible for rhizosheath formation.

Some genes such as *TaALMT1* (aluminium‐activated malate transporter) may assist rhizosheath formation by secreting malate, thereby conferring Al‐resistance and protecting fine and coarse root elongation allowing root development in acidic soils, while Al‐sensitive lines showed reduced rhizosheath mass and root growth in wheat (Delhaize et al., 2015). In *Sorghum bicolour*, the *SbMATE* (multidrug toxic compound extrusion) involved in root growth processes (Carvalho et al., 2016) secretes organic acid (citrate), which has been hypothesized as a potent stimulator of soil microbial activity (Macias‐Benitez et al., 2020). However, a recent study identified 12 potential QTLs may control rhizosheath formation in pearl millet, indicating complex genetic regulation mainly via root exudation (De la fuente Cantó et al., 2022). Therefore, these genes/QTLs associated with root growth and stress‐responsiveness may improve abiotic stress tolerance. Taken together, these findings suggest various genes (Table 1) that are functionally significant in rhizosheath formation via mechanisms attributed to root growth and organic acid exudation, but their effects vary between species and rhizosheath formation is a multi‐genic phenomenon.

**Table 1:** Genes/QTLs related to rhizosheath formation in different crop species

Plant	Gene/QTLs	Role in rhizosheath formation	References
Transgenic barley	Overexpressed with wheat gene *TaALMT1*	Enhanced malate efflux, efficient P uptake and grain production.	Delhaize et al. (2009)
Barley	QTL on chromosome 2H	Rhizosheath weight correlated with P uptake under dry conditions	George et al. (2014)
Pearl millet	Twelve QTLs	Rhizosheath formation was complex, mainly regulated by root exudation	De la fuente Cantó et al. (2022)
Foxtail millets	*Seita.3G196500, Seita.9G333500, Seita.8G104600, Seita.2G057800* and *Seita.7G190800*	Showed higher transcript level under drought conditions	Liu, Ye et al. (2019)
Rice	*L. albABCG29*	Improved P use through enhanced root growth and increased rhizosheath formation under low P soil drying	Aslam, Wassem et al. (2021)
Wheat	Chromosome no 2B, 4D, 5A, 5B, 6A and 7A and five major loci	Contribute to 42% rhizosheath variations, accounting for over 60% of the total genetic variance	Delhaize et al. (2015); James et al. (2016); Marr (2021)

Abbreviation: Quantitative trait loci.

### 2.2 Physico‐chemical factors

Drying and wetting cycles promote soil aggregation by modifying the soil pore system, with soil pores being filled with water during wetting, and irreversible rearrangement of soil particles during drying (Pires et al., 2007). This process interacts with other gluing agents released in the root‐zone by the roots themselves or soil microbes, playing complementary roles in promoting soil particle aggregation and adhesion to the root surface (rhizosheath formation). Drier soils enhanced adhesiveness of the rhizosheath and facilitated root hair formation; both of which are involved in rhizosheath stabilisation (Watt et al., 1994).

Soil characteristics such as texture, aggregate stability, organic matter content, number of drying and rewetting cycles, and soil water content all affect rhizosheath stability (Ghezzehei & Albalasmeh, 2015; Haling et al., 2010; Haling et al., 2014). Roots growing in soils with higher (80%) sand content had more root hairs and more pronounced rhizosheaths than those growing in lower (30%) sand content (Bailey & Scholes, 1997). Barley genotypes (differing in root hair development) had greater rhizosheath development when grown in a sandy loam than a clay loam (Marin et al., 2021). When grown in a sandy loam soil compacted to a range of bulk densities (1.2−1.7 g cm^−3^), barley rhizosheath development was greatest at the lowest bulk density (1.2 g cm^−3^) but declined at next highest (1.4 g cm^−3^) despite similar root hair lengths, indicating the importance of both soil physical and chemical processes (Haling et al., 2014). Fine textured soils were predicted to produce more water stable aggregates at a lower soil organic matter content when exposed to drying and rewetting cycles (Albalasmeh & Ghezzehei, 2014). Thus physical and chemical processes interact in rhizosheath formation.

Rhizosheath formation alters soil particle size distribution around the roots, increasing the frequency of smaller particles compared to the bulk soil (Wei et al., 2011). Water fluxes associated with plant transpiration move colloidal particles within the soil towards the roots (Albalasmeh & Ghezzehei, 2014), while disaggregation of soil particles in response to the exudation of organic acids also contributes (Naveed et al., 2017). The size of soil pores relative to root hair diameter likely determines the importance of physical versus chemical processes in determining root adhesion to the surrounding soil. In dense soils, roots encounter series of channels, and biopores, or grow between regions of soil of contrasting strength. More than 85% of wheat roots deep in the soil profile (below 50 cm) were clumped within biopores and channels (White & Kirkegaard, 2010), with substantial root hair proliferation along the pore wall acting to maintain hydraulic root‐soil contact. Moreover, root hairs may contribute to anchorage to assist the penetration of individual root tips and increase the soil–root binding (De Baets et al., 2020).

Alternate wetting and drying cycles (driven by evapotranspiration) interact with mucilage (a viscoelastic gel substance rich in polysaccharides) at the root‐soil interface to stimulate soil structure changes and increase rhizosheath porosity, as determined using microcomputed tomography (Y. Zhang, Du, Gui, et al., 2020). The quantity and quality of polysaccharides, and the ambient soil matric potential both play an important role in the mucilage's ability to aggregate soil. Mucilage greatly affects soil water dynamics. During soil drying, it sustains higher soil water content which potentially enhances soil aggregation, but at lower water potentials mucilage becomes hydrophobic and decreases rhizosphere hydraulic conductivity (Kroener et al., 2014; Rosenzweig et al., 2009). With prolonged soil drying, mucilage is suggested to lose water to the surrounding soil (Ahmed et al., 2014), thereby increasing its viscosity and in turn decreasing its surface tension. Decreased surface tension increases their ability to wet the surrounding soil (Read & Gregory, 1997), while high viscosity of exudates increases the resistance of adhering soil particles to movement (Moradi et al., 2012; Read & Gregory, 1997). Consequently, rhizosheath stability and thickness increases (Walker et al., 2003). Interestingly, the rhizosheath of drought‐tolerant grasses (growing in the African savannah) was significantly (*p* < 0.0001) thicker (by 65%) than in drought‐sensitive grasses (Hartnett et al., 2013), suggesting that the rhizosheath enhances rhizosphere water retention capacity under drought stress, thereby promoting plant growth and survival.

Exudates/mucilage enable plants to create microenvironments which can be beneficial for plant growth. The release of polysaccharide‐rich mucilage from the root tips eases root penetration in deeper soil layers and may serve as a protective barrier (Galloway et al., 2020). Since microbial and root contributions to rhizosheath development can be difficult to distinguish, future experiments should aim to exclude the confounding effects of microbial community‐derived mucilage secretions. Plants could be grown in clean river sand as it has low microbial activity (Marasco et al., 2018; Neilson et al., 2017) or soils could be sterilized by gamma irradiation or other ways (Cheptsov et al., 2021; Mahmood et al., 2014; Yin & Wang, 2016). Such practices may better distinguish the relative contribution of root versus microbial mucilage to rhizosheath formation.

### 2.3 Microbial community

Severe drought stress decreases the size and activity of rhizosphere microbial biomass (Sanaullah et al., 2011). Microbes have adopted various strategies to thrive in the rhizosphere niche (Aslam et al., 2022; Jacoby et al., 2017). Many bacterial genera release exopolysaccharides (EPS) (Costa et al., 2018) including *Bacillus, Pseudomonas* and *Azospirillum brasilense*, which permeate the surrounding soils bonding them together and increasing their aggregation to the root surface as mucilage dries (Walker et al., 2003). Soil drying stimulates EPS production, thereby enhancing soil moisture retention and buffering bacterial colonies from environmental stress (Roberson & Firestone, 1992). Soil inoculation with EPS‐producing bacteria, that produce more EPS as the soil dries, can enhance soil aggregation and rhizosheath development, thereby improving leaf water status when plants are grown in drying soil (Alami et al., 2000; Sandhya et al., 2009). Thus, it has been postulated that rhizosheath‐associated microbial communities play a profound role in forming a coherent rhizosheath (Mccully & Canny, 1988; Watt et al., 1993).

Certain classes of soil microbes released volatile organic compounds (including esters, ethers, aldehydes, naphthyl derivatives, ketones, alkyls and benzene derivatives) which facilitate microbial interactions (Yuan et al., 2017) and induce root secretion (Phillips, 2007; Phillips et al., 2008) or modification (Fernandez et al., 2016) of plant metabolites such as organic acids, which greatly influence rhizosheath formation. Bacterial exudation of various compounds can alter rhizosheath water holding capacity during soil drying. Wheat plants inoculated with *Bacillus* (T‐34) and *Azospirillum* (WS‐1) strains promoted root growth and increased rhizosheath dry weight compared to non‐inoculated plants under both wheat‐cotton and wheat‐rice crop rotations (Tahir et al., 2015). Inoculating EPS‐producing bacteria could maintain soil water content to alleviate drought stress (Ashraf et al., 2004), suggesting these beneficial bacteria could serve as a promising strategy to alleviate abiotic stresses. Optimal water content around the root is associated with increased enzymatic activity, typically phosphatase activity (Guenet et al., 2012), which assists phosphate acquisition in plants, consequently promoting crop growth (Hu et al., 2013; Turan et al., 2012). However, it can be difficult to determine the role of specific microbial traits in enhancing rhizosheath development and plant stress tolerance, as many bacteria have multiple beneficial traits. For example, EPS‐producing bacteria can also produce ammonia, hydrogen cyanide and the phytohormone indole 3‐acetic acid (Khan & Bano, 2019), all of which might affect root and rhizosheath development in drying soil. More specific evidence on the importance of microbial EPS in rhizosheath development should be sought by obtaining bacterial mutants that lack EPS production (Deka et al., 2019) and determining their impact when plants are grown in drying soil.

A recent example utilized bacterial mutagenesis to demonstrate the impacts of particular microbial traits on rhizosheath development (Y. Zhang, Du, Xu et al., 2020). Inoculating rice with the aminocyclopropane‐1‐carboxylic acid (ACC) deaminase containing rhizobacterium *Enterobacter aerogenes* G3 almost doubled rhizosheath formation compared to its ACC‐deaminase minus mutant, presumably since the WT strain produced significantly longer root hairs, physically enmeshing the soil. Surprisingly, previous in vitro investigations with a range of WT rhizobacteria and their ACC‐deaminase minus mutants indicated that the latter produced significantly longer root hairs (Contesto et al., 2008), although the effects of perturbing root ethylene status on root hair development will depend on other microbially produced hormones and the root growth environment. Alternatively, adding the ACC deaminase gene to *A. brasilense* increased root hair density of *Vicia sativa* (Star et al., 2012), which should promote rhizosheath development. Interestingly, the rice rhizosheath was enriched with *Enterobacteriaceae* (Y. Zhang, Du, Xu et al., 2020), leading to the hypothesis that crop species may select specific taxa of beneficial soil microbes to improve water status through unidentified mechanisms, presumably via signals derived from root exudates. Whether there are genetic determinants that underpin how different plant species interact with rhizospheric microbial communities needs attention, as such discoveries may provide potential targets for breeding water efficient crops.

## 3 RHIZOSHEATH INCREASES PHOSPHORUS UPTAKE **BY** PLANT ROOTS

Phosphorus is an essential macronutrient for crop growth and yield, yet it is poorly available in the soil for plant uptake owing to its low diffusion rate (Lambers et al., 2015, 2018). To cope with P deficiency and support growth‐related processes, plants forage the soil for this sparingly available resource, by enhancing lateral root branching and root hair development (Lambers et al., 2015; Lynch & Brown, 2001; Lynch, 2019). Long root hairs increase the surface area for intercepting P diffusing towards the roots (Haling et al., 2016). This root‐hair‐cylinder, in most cases, positively correlates with rhizosheath size (Haling et al., 2010). Enhanced rhizosheath development alleviated P deficiency stress of barley and wheat (Brown et al., 2012; Haling et al., 2013). Hence, while rhizosheath formation and root hair growth are correlated with enhanced P uptake efficiency, other mechanisms by which rhizosheath facilitates uptake of P also exist.

**Figure 2:**
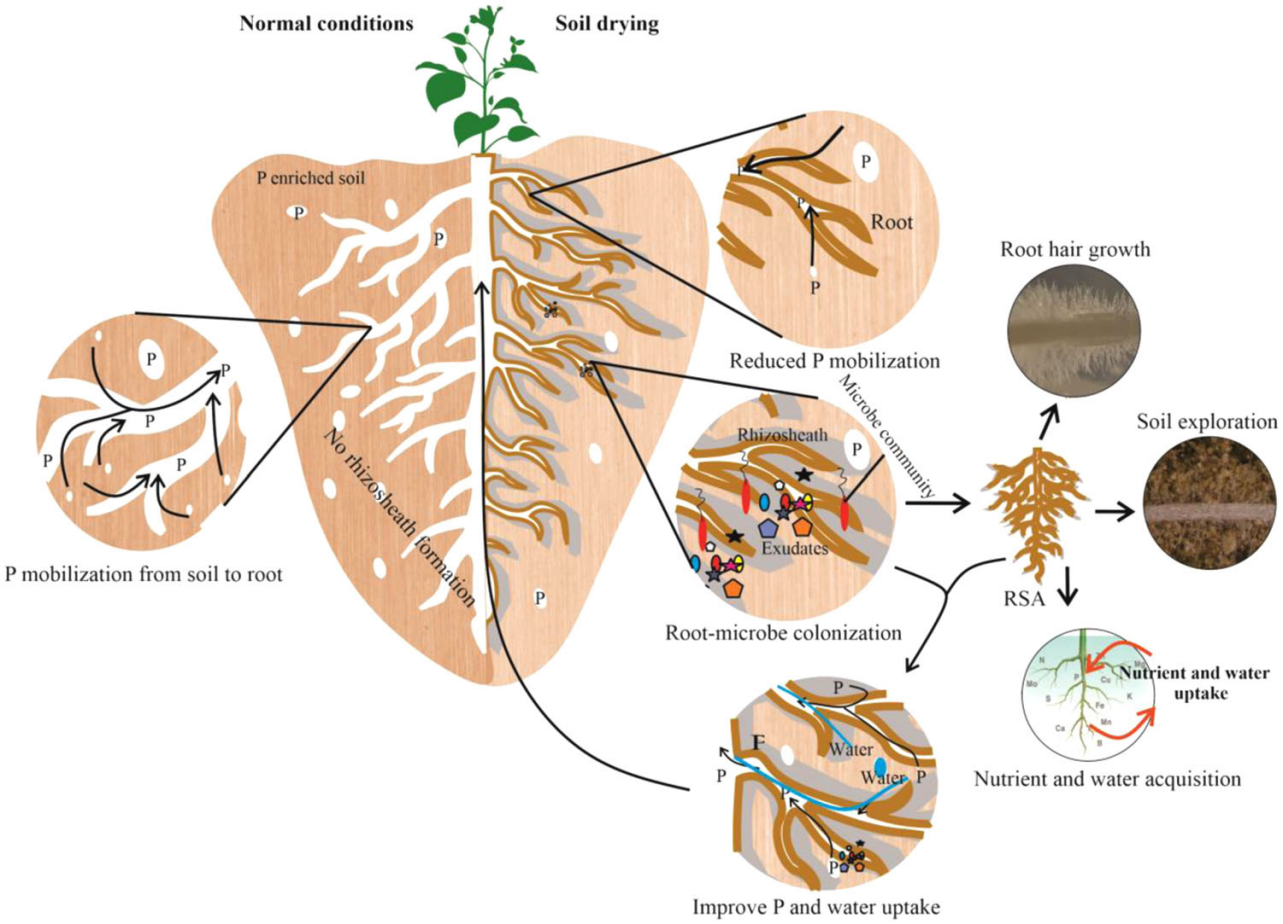
Soil drying and its effects on plant tissue P concentrations. Soil drying events activate root exudation which in turn enhances rhizosheath formation. Root exudates mediate P‐deprivation signalling, ultimately altering RSA which improves soil exploration for nutrient and water uptake. Additionally, root exudation initiates root microbial colonisation in the rhizosphere. Together, organic acids exuded by plant roots and microbes promote phosphorus solubilisation. RSA, Root system architecture; P, phosphorus. [Color figure can be viewed at wileyonlinelibrary.com]

In P‐efficient genotypes growing in low P availability, plant roots release exudates to mobilize nutrients in the rhizosphere (Krishnapriya & Pandey, 2016; Nazari, 2021). Components of these root exudates help plants to access nutrients by acidifying the rhizosphere or chelating nutrients (Raghothama, 1999). Oat (*Avena sativa*) and sugar beet (*Beta vulgaris*) plants were grown in roots split between soil compartments, to expose part of the root system to dry soil while keeping the other soil compartment moist to avoid leaf water deficit. Although decreased K inflow was consistent with a modelled diffusional impediment, soil moisture content did not influence P inflow due to increased root exudation rate, especially of high molecular weight compounds from water‐stressed plants, which increased P concentrations in the soil solution (Liebersbach et al., 2004). Thus, exudation of mucilage could be a promising route to overcome P uptake and transport under soil drying events (Figure 2).

Malate, citrate, and malonate are potent organic acids exuded by various crop species. Carboxylate (malonate and citrate) secretion by legumes and white lupin solubilizes soil P, making it accessible for roots to capture (Dinkelaker et al., 1989; Hocking et al., 1997; Kidd et al., 2016). The lupin rhizosheath had a higher carboxylate content than those of pasture legumes such as *Trifolium* and *Medicago* species (Kidd et al., 2016), suggesting that lupin may better mobilize minimally available P from the rhizosphere. Thus, P homoeostasis likely involves complex and highly coordinated processes with contributions potentially from root system development and physico‐chemical components of root exudates and microbial secreted mucilage (Figure 2). Cover crop (*Sinapis alba, Phacelia tanacetifolia*, and *Fagopyrum esculentum*) rhizosheath soil had higher microbial abundance than the bulk soil (Hallama et al., 2022), which was partially attributed to rhizodeposition (presumably organic acids) providing a C‐source for microbes associated with P cycling (M. S. Khan et al., 2014) which can enhance availability of P for plant use (Richardson & Simpson, 2011). This is likely since plant roots exude organic acids and extracellular enzymes (phytase and acid phosphatase) to mobilise inorganic P from Al, Ca or Fe phosphates (Felix & Donald, 2002), or organic P compounds (Chen et al., 2006; J. F. Ma et al., 2001), allowing absorption by the plant. Upon P deficiency, plants release specific root exudates like gamma‐aminobutyric acid (GABA, acting as a chemo‐attractant for P‐solubilizing microbes), and acidify the rhizosphere to make additional Pi available (Dakora & Phillips, 2002; Felix & Donald, 2002; Hinsinger, 2001). These processes ultimately improve nutrient acquisition and availability in nutrient‐depleted soils.

Rhizosphere bacteria can independently (Jeong et al., 2013; Qureshi et al., 2012; Scagliola et al., 2016) or via interaction with plants (Pii et al., 2015; Segura & Ramos, 2013), secrete P‐solubilizing enzymes and organic acids, thus promoting absorption of previously insoluble nutrients by plant (Pii et al., 2015, 2016; Rodriguez et al., 2004). However, how root exudates reprogramme the root microbiome (Pascale et al., 2020) is still not clear. Cover crops can increase soil P availability by enhancing the abundance and activity of P‐solubilising microbes in the rhizosheath (Hallama et al., 2022). Although integrative analyses of microbial composition and microbial functional genes associated with P cycling remain significantly challenging owing to variability of P in the field, they may play a crucial role in P acquisition in the rhizosheath. Similarly, high rhizosheath phosphatase activity has been associated with enhanced P acquisition, thereby increasing root and shoot biomass, and P accumulation (Hunter et al., 2014). Since rhizodeposition by the root system nourishes the rhizobiome and promotes rhizosheath formation, further studies determining expression of rhizosheath phosphatase activity genes would extend our understanding of rhizosheath‐dependent P acquisition.

## 4 RHIZOSHEATH IMPACTS ON PLANT WATER UPTAKE

Empirical evidence demonstrates that soil moisture in the rhizosheath was substantially higher than that of the surrounding bulk soil in different plant species, according to the absolute soil water potential (Moradi et al., 2012; Young, 1995). While wheat and barley roots had hydrophilic mucilage (Naveed et al., 2017; Zickenrott et al., 2016), some rhizosheaths were more hydrophobic than others (Brown et al., 2017), with maize, lupins and chia roots having hydrophobic mucilage (Zeppenfeld et al., 2017). Furthermore, a modelling exercise (Carminati et al., 2011) showed that mucilage attenuates gradients in water potential in the rhizosphere, thus increasing water acquisition by roots as the soil dries.

Rhizosheath formation can act as a ‘bridge’ to the bulk soil, thereby minimising the formation of air gaps adjacent to the root system, which substantially increase hydraulic resistance at the soil‐plant interface (North & Nobel, 1997). Mucilage exuded by the roots limits the development of air gaps around roots, and improves the ability of plant roots to take up water by decreasing hydraulic resistance in the rhizosphere (Ahmed et al., 2014; Carminati et al., 2014). Nevertheless, analysing root shrinkage (air gap formation) in relation to soil water status as broad bean (*Vicia faba*) and white lupin plants dried a sandy soil (Carminati & Vetterlein, 2013; Koebernick et al., 2018) demonstrated that root shrinkage occurred after transpiration rate declined, suggesting that other factors than root‐soil contact were limiting water uptake.

While air gaps may restrict root water uptake from the soil, they may also reduce water loss from roots towards the soil (Carminati & Vetterlein, 2013; Zarebanadkouki & Carminati, 2014) which becomes important when soil water potential is lower than root water potential. Although mucilage secretion may initially hydrate the rhizosheath, it may later limit water loss as soil drying progresses (Ahmed et al., 2014; Carminati & Vetterlein, 2013). The gelling effect of mucilage within the rhizosheath can decrease water flow from the rhizosheath soil to the bulk soil distant from the root surface, presumably by reducing the matric potential gradient between rhizosheath and the bulk soil (Ahmed et al., 2016; Carminati et al., 2016). Prolonged soil drying can make the mucilage water‐repellent (Czarnes et al., 2000; Lichner et al., 2002; Moradi et al., 2012), thus limiting plant water uptake when the soil is re‐watered, and delaying the recovery of leaf water potential and stomatal conductance. Thus the adaptive value of mucilage will vary according to the severity and frequency of drying episodes, and its chemical composition.

Several studies have compared the responses of mutants lacking root hairs with their respective WT, although effects of root hairs *per se*, rather than their contribution to rhizosheath formation, cannot be distinguished. When the bald root barley (*brb*) mutant and its WT were exposed to abrupt changes in evaporative demand, the mutant could not sustain transpiration rates at high vapour pressure deficits (VPDs) without substantially decreasing leaf water potential (Carminati et al., 2017). At more moderate VPDs (<1.5 kPa), there were no genotypic differences in transpiration rate, leaf water potential or xylem ABA concentration (Dodd & Diatloff, 2016), indicating that root hairs (and presumably rhizosheath formation) were redundant in sustaining water uptake. Alternatively, wheat cultivars with larger rhizosheaths sustained higher transpiration rates as the soil dried, while contrasting cultivars with diminished rhizosheath wilted more readily (Basirat et al., 2019). Further work is required using imaging techniques (X‐ray Computer Tomography and magnetic resonance imaging) to determine the timing of air gap formation relative to the decline in transpiration in genotypes that differ in rhizosheath development. To be confident of rhizosheath impacts on plant water relations, future work should aim to manipulate rhizosheath formation via different mechanisms (not just variation in root hair development) to determine whether physiological responses to variation in rhizosheath development are consistent.

## 5 ALTERNATE WETTING AND DRYING OF RICE TO ENHANCE WATER AND P USE EFFICIENCY

Alternate drying and rewetting cycles (ADW) is a deliberate strategy used to save water in agriculture, thereby improving crop water use efficiency (Dodd et al., 2015). Repeated ADW cycles in rice paddy fields dynamically alters the microbial community, soil water and nutrient availability (Watanabe et al., 2021). Allowing rice fields to periodically dry out by withholding irrigation can decrease water use by 20%−40% compared to continuous flooding, without influencing rice production or yield (Carrijo et al., 2017; Rejesus et al., 2011). While part of this water saving will occur through physical processes (decreased evaporation and drainage/leakage from rice paddies), stomatal closure can also occur if soil matric potential declines sufficiently (Li et al., 2017). Such stomatal closure cannot always be attributed to decreased leaf water potential (Dingkuhn et al., 1989; X. Wang et al., 2018), but instead to the root‐to‐shoot transport of chemical signals like ABA (Bano et al., 1993; Siopongco et al., 2008). Irrespective of the mechanisms of stomatal closure, intrinsic water use efficiency increases (Caine et al., 2019), since transpiration is restricted more than photosynthesis (Hidayati & Anas, 2016). Selecting genotypes with variation in the production of, or sensitivity to, root‐to‐shoot signals (especially ABA) under progressive soil ADW or drought may enhance crop water use efficiency and sustain yields under environmental stresses, although it is less clear that these signalling processes are related to rhizosheath development.

Multiple mechanisms inhibit plant growth as the soil dries, including decreased P uptake and transport (Rouphael et al., 2012). Upon rewetting, physico‐chemical processes that disrupt dried soil aggregates release P into the soil solution (Bünemann et al., 2013), along with lysis of microbial cells associated with osmotic shock (Blackwell et al., 2010; Bünemann et al., 2013; Turner et al., 2003). Thus, the impacts of ADW cycles (especially in periodically flooded soils such as in rice cultivation) on plant P uptake can be difficult to predict, because these different microbial and physico‐chemical processes affect soil P availability. Repeated ADW events confer resistance to some microbial communities rendering them less susceptible to osmotic stress‐induced lysis (Butterly et al., 2009; Sawada et al., 2016). The frequency of ADW can influence the microbial community composition, with microbes capable of recovering from desiccation sustaining the microbial community (Zhao et al., 2016) and solubilizing P allowing plant uptake. Both ADW events (Denef et al., 2001), and root mucilage (Chaparro et al., 2014; Haichar et al., 2012; Kawasaki et al., 2016; Terrazas et al., 2016) can select for specific microbial communities that are tolerant to rewetting stresses or communities that are specific to plant species, respectively. Possibly rhizosheath formation sustains these microbial communities, helping plants to cope with nutrient and water constraints.

Whether ADW contributes to rhizosheath formation to enhance P use efficiency of rice is still uncertain, especially since it may be difficult to measure rhizodeposition (Kuzyakov & Domanski, 2000) and nutrient concentrations in the soil solution (Norton et al., 2017) in such systems. Since root exudates involved in rhizosheath formation are a crucial adaptive strategy for plant P acquisition from P‐deficient soils (Ndour et al., 2020), such findings imply that ADW may potentially contribute to P uptake via rhizosheath formation. Experimental evidence of precise mechanisms underlying the complementary roles of ADW and rhizosheath formation in determining water and P use efficiency is still scarce.

## 6 CONCLUSIONS AND FUTURE PERSPECTIVES

Rhizosheath formation requires complex interactions of root hair traits with root‐ and microbe‐derived mucilage. To assist plant breeding efforts, it is imperative to uncover the underlying genetic mechanism(s) that promote efficient water and phosphorus use under suboptimal soil conditions, and identifying genes regulating rhizosheath formation may be important in this endeavour. While most work demonstrating the potential importance of rhizosheath development in regulating plant physiological responses to soil conditions has compared WT plants and mutants lacking root hairs, exploiting genetic diversity in root hair traits and root exudation profiles within diverse germplasm of major crop species seems important. Since these inter‐connected physical and chemical plant‐mediated processes interact with multiple soil properties in determining rhizosheath formation, pronounced genotype × environment interactions are highly likely. Whether plant breeders developing crop genotypes adapted to water‐ and nutrient‐limited environments have unconsciously selected for enhanced rhizosheath formation in specific environments remains to be determined, but enhancing these processes may be critical to further progress in improving crop resilience to these stresses.

The development of new techniques to discriminate genetic variation in processes affecting rhizosheath development will complement traditional rhizosheath measurements that excavate the root system and quantify the adhering soil. The ability of root exudates to bind soil *ex vivo* can be determined by collecting them from hydroponically grown plants, concentrating them and then applying them to nitrocellulose. Dried, sieved soil is then placed on the membrane and allowed to adhere, with unbound soil removed before quantifying that remaining on the membrane (Akhtar et al., 2018). This technique discriminated variation in exudate adhesiveness between WT and root hairless mutants (Burak et al., 2021), but has yet to be applied to large populations of genetically related plants such as recombinant inbred lines to identify potential QTLs mediating rhizosheath development. This soil‐based technique complements high‐throughput antibody‐based tests such as ELISA (Galloway et al., 2020) that can measure root polysaccharide release in the same hydroponic samples. However, the impact of the soil environment (moisture and P status) on root exudation requires further investigation, to ensure that these ex vivo assays correlate with in vivo rhizosheath development.

While it is clearly evident that stimulation of rhizosheath development in drying soil is dependent on root hair traits and root exudation, further work focusing on the association between root exudate chemistry and expression profiles of P‐uptake genes is warranted. Moreover, further exploration of the association between rhizosheath traits (weight, strength and porosity) and P uptake are necessary in crop species grown under varied soil types and contrasting water levels. Such studies would help define the utility of rhizosheath in crop breeding for greater sustainability.

## CONFLICT OF INTEREST

The authors declare no conflict of interest.
